# Pseudomonas Aeruginosa Endocarditis in Acute Myeloid Leukemia: A Rare Complication

**Published:** 2008-12

**Authors:** Barshay J, Nemets A, Ducach A, Lugassy G

**Affiliations:** *Department of Hematology, Barzilai Medical Center, Ashkelon, Affiliated to the Ben Gurion University of the Negev, Beer Sheva, Israel*

**Keywords:** endocarditis, leukemia, platelets

## Abstract

Infectious endocarditis is a rarely encountered complication among leukemia patient during induction therapy. We describe a young patient who developed prolonged high fever after aggressive chemotherapy for Acute Myeloid Leukemia. Pseudomonas Aeruginosa endocarditis was found to be the etiology for the febrile state. Our purpose is to emphasize the need for an early diagnosis of this rare, albeit treatable complication.

## INTRODUCTION

Severe bacterial infections are one of the most important causes of morbidity and mortality encountered among acute leukemia patients. They primarily occur during induction therapy and may increase risk of leukemia relapse (1). Although Gram positive infections are more frequent, morbidity and mortality are usually associated with Gram negative or deep fungal infections (2). Endovascular catheterization is the main origin of bacteremia in acute leukemia patients, especially with gram-positive microorganisms.

We describe an AML patient who developed Gram negative, *Pseudomonas Aeruginosa*, right sided endocarditis, probably associated with central line access.

A previously healthy 57 year old man was admitted to the department of hematology with a one week history of fever and severe necrotizing cellulitis.

He was a former 20 pack-year cigarette smoker and received radiotherapy because of lichen plaques some 50 years ago.

On admission he appeared tired and pale, with a fever of 38.5°C. There was no hepatosplenomegaly or lymph nodes enlargement. Severe swelling and redness were present on his right arm and leg, with black blisters on his right foot and fingers.

The x-ray chest examination was normal. Blood analyses revealed severe thrombocytopenia of 36 × 10^9^/l. WBC were -5.59 × 10^9^/l, with 31% neutrophils, 32% lymphocytes and 37% monocytes. Hemoglobin level was 11.2 g/dl.

A peripheral blood examination revealed 70% blast cells, and a bone marrow examination showed a massive infiltration with myeloid blasts. Cytogenetic analysis revealed a translocation t (1:10)

Renal and liver tests were within normal range. Serum glucose levels were elevated, with no metabolic acidosis.

Therapy with broad-spectrum antibiotics (piperacillin/tazobactam), fluids and regular insulin subcutaneously was initiated.

A central venous catheter was inserted and induction chemotherapy was initiated (7 day ARA-C-100 mg/m^2^/day + 3 day doxorubicin 45mg/m^2^/day intravenously).

A complete remission (CR) was achieved after the second course of induction chemotherapy.

Two months later the patient began to suffer from episodes of high temperature up to 40°C. Blood cultures, performed on Mac Conkey agar plate at 42°C for 24 hours, demonstrated the growth of Gram-negative bacilli's (*Pseudomonas Aeruginosa*).

Despite intensive management of bacteremia by combination antibacterial therapy (tazocin+amykacin), *Pseudomonas* was still present in repeated blood cultures, and the central venous catheter was removed.

Transthoracic echocardiography and transesophageal echography (TEE) revealed three vegetations on the tricuspid valve without damage of the valve or tricuspid regurgitation.Intravenous Imipenem was added to the antibiotic regimen for six more weeks. The patient was then discharged in good clinical condition with normal blood counts.

Five days after discharge, high fever recurred, accompanied with chills. A profound pancytopenia was present but a bone marrow aspiration showed no relapse of AML.

Broad spectrum antibiotic regimen was restarted. GCS-F therapy was added (3). A CT-scan of the abdomen revealed no abscess. A second TEE revealed two vegetations on the tricuspid valve and one on the pulmonary valve, without ring abscess or marked regurgitation. Blood cultures were still positive for *Pseudomonas Aeruginosa*.

Combined antibiotherapy was continued for another eight weeks. One month after discharge, pancytopenia was still severe, but a bone marrow examination showed no evidence of acute leukemia. The patient died one month later from fulminant Candida sepsis unresponsive to therapy.

## DISCUSSION

Systemic infections represent the most frequent complications of chemotherapy for acute myeloid leukemia patients and are associated with high morbidity and mortality.

Gram negative bacterial infections, are the most frequent and life threatening complications in this population (4, 6).

*Pseudomonas Aeruginosa* is a common nosocomial contaminant in hospitalized patients and is responsible for the occurrence of severe infections such as malignant external otitis, endophtalmitis, endocarditis, meningitis and pneumonia (5).

Surprisingly, infectious endocarditis is a complication rarely encountered among patients treated for acute leukemias (8-10), the reasons why are not clear.

All conditions for the development of infectious endocarditis do exist among patients treated for leukemia’s:

- Endothelial dysfunction due to the use of aggressive chemotherapy regimen;
- Immunosupression secondary to the disease and its therapy;
- Presence of many “porte of entry” for infectious agents: chronic indwelling catheters (7), wounds, etc.

Intracardiac vegetations are the results of fibrin deposition combined with platelet aggregation, stimulated with tissue factor and by proliferating organisms. In the absence of an adequate immune system, organisms' emmeshed in the growing platelet-fibrin vegetations proliferate to form dense micro colonies.

The chronically low platelet count seen in leukemic patients, secondary to both the hematological disorder and its aggressive therapy, may indeed be the cause for the low frequency of endocarditis encountered in this population.

Another possible explanation for the rarity of this diagnosis is the fact that most leukemic patients have several causes for their high fever and that endocarditis may be overlooked after a reasonable etiology is found to explain the fever and the signs of systemic infection.

We describe an AML patient who developed *Pseudomonas Aeruginosa* right sided endocarditis, and responded well to prolonged systemic combined antibiotherapy.

To our knowledge, this is the first reported case of *Pseudomonas Aeruginosa* tricuspid and pulmonic valves endocarditis complicating chemotherapy in a leukemic patient.

Infectious endocarditis may be an overlooked diagnosis among treated leukemic patients. Its early diagnosis and treatment may improve the outcome of such patients.
